# Clinicogenomic predictors of outcomes in patients with hepatocellular carcinoma treated with immunotherapy

**DOI:** 10.1093/oncolo/oyae110

**Published:** 2024-06-27

**Authors:** Darren Cowzer, Joanne F Chou, Henry Walch, Fergus Keane, Danny Khalil, Jinru Shia, Richard K G Do, Hooman Yarmohammadi, Joseph P Erinjeri, Imane El Dika, Amin Yaqubie, Hassan Azhari, Maya Gambarin, Carla Hajj, Christopher Crane, Alice C Wei, William Jarnagin, David B Solit, Michael F Berger, Eileen M O’Reilly, Nikolaus Schultz, Walid Chatila, Marinela Capanu, Ghassan K Abou-Alfa, James J Harding

**Affiliations:** Department of Medicine, Memorial Sloan Kettering Cancer Center, New York, NY, United States; Department of Epidemiology and Biostatistics, Memorial Sloan Kettering Cancer Center, New York, NY, United States; Kravis Center for Molecular Oncology, Memorial Sloan Kettering Cancer Center, New York, NY, United States; Department of Medicine, Memorial Sloan Kettering Cancer Center, New York, NY, United States; Department of Medicine, Memorial Sloan Kettering Cancer Center, New York, NY, United States; Weill Medical College of Cornell University, New York, NY, United States; Weill Medical College of Cornell University, New York, NY, United States; Department of Pathology, Memorial Sloan Kettering Cancer Center, New York, NY, United States; Weill Medical College of Cornell University, New York, NY, United States; Department of Radiology, Memorial Sloan Kettering Cancer Center, New York, NY, United States; Weill Medical College of Cornell University, New York, NY, United States; Department of Radiology, Memorial Sloan Kettering Cancer Center, New York, NY, United States; Weill Medical College of Cornell University, New York, NY, United States; Department of Medicine, Memorial Sloan Kettering Cancer Center, New York, NY, United States; Weill Medical College of Cornell University, New York, NY, United States; Department of Medicine, Memorial Sloan Kettering Cancer Center, New York, NY, United States; Department of Medicine, Memorial Sloan Kettering Cancer Center, New York, NY, United States; Weill Medical College of Cornell University, New York, NY, United States; Department of Medicine, Memorial Sloan Kettering Cancer Center, New York, NY, United States; Weill Medical College of Cornell University, New York, NY, United States; Weill Medical College of Cornell University, New York, NY, United States; Department of Radiation Oncology, Memorial Sloan Kettering Cancer Center, New York, NY, United States; Weill Medical College of Cornell University, New York, NY, United States; Department of Radiation Oncology, Memorial Sloan Kettering Cancer Center, New York, NY, United States; Weill Medical College of Cornell University, New York, NY, United States; Department of Surgery, Memorial Sloan Kettering Cancer Center, New York, New York, NY, United States; Weill Medical College of Cornell University, New York, NY, United States; Department of Surgery, Memorial Sloan Kettering Cancer Center, New York, New York, NY, United States; Department of Medicine, Memorial Sloan Kettering Cancer Center, New York, NY, United States; Kravis Center for Molecular Oncology, Memorial Sloan Kettering Cancer Center, New York, NY, United States; Weill Medical College of Cornell University, New York, NY, United States; Kravis Center for Molecular Oncology, Memorial Sloan Kettering Cancer Center, New York, NY, United States; Weill Medical College of Cornell University, New York, NY, United States; Department of Pathology, Memorial Sloan Kettering Cancer Center, New York, NY, United States; Department of Medicine, Memorial Sloan Kettering Cancer Center, New York, NY, United States; Weill Medical College of Cornell University, New York, NY, United States; Kravis Center for Molecular Oncology, Memorial Sloan Kettering Cancer Center, New York, NY, United States; Weill Medical College of Cornell University, New York, NY, United States; Department of Pathology, Memorial Sloan Kettering Cancer Center, New York, NY, United States; Kravis Center for Molecular Oncology, Memorial Sloan Kettering Cancer Center, New York, NY, United States; Department of Epidemiology and Biostatistics, Memorial Sloan Kettering Cancer Center, New York, NY, United States; Department of Medicine, Memorial Sloan Kettering Cancer Center, New York, NY, United States; Weill Medical College of Cornell University, New York, NY, United States; Department of Medicine, Memorial Sloan Kettering Cancer Center, New York, NY, United States; Weill Medical College of Cornell University, New York, NY, United States

**Keywords:** immunotherapy, immune checkpoint inhibitors, biomarkers, tumor

## Abstract

**Introduction:**

Immune checkpoint inhibitor (ICI) combinations extend overall survival (OS) while anti-PD-1/L1 monotherapy is non-inferior to sorafenib in treatment-naïve, patients with advanced hepatocellular carcinoma (HCC). Clinicogenomic features are posited to influence patient outcomes.

**Methods:**

The primary objective of this retrospective study was to define the clinical, pathologic, and genomic factors associated with outcomes to ICI therapy in patients with HCC. Patients with histologically confirmed advanced HCC treated with ICI at Memorial Sloan Kettering Cancer Center from 2012 to 2022 were included. Association between clinical, pathological, and genomic characteristics were assessed with univariable and multivariable Cox regression model for progression-free survival (PFS) and OS.

**Results:**

Two-hundred and forty-two patients were treated with ICI-based therapy. Patients were predominantly male (82%) with virally mediated HCC (53%) and Child Pugh A score (70%). Median follow-up was 28 months (0.5-78.4). Median PFS for those treated in 1st line, 2nd line and ≥ 3rd line was 4.9 (range: 2.9-6.2), 3.1 (2.3-4.0), and 2.5 (2.1-4.0) months, respectively. Median OS for those treated in 1st line, 2nd line, and ≥ 3rd line was 16 (11-22), 7.5 (6.4-11), and 6.4 (4.6-26) months, respectively. Poor liver function and performance status associated with worse PFS and OS, while viral hepatitis C was associated with favorable outcome. Genetic alterations were not associated with outcomes.

**Conclusion:**

Clinicopathologic factors were the major determinates of outcomes for patients with advanced HCC treated with ICI. Molecular profiling did not aid in stratification of ICI outcomes. Future studies should explore alternative biomarkers such as the level of immune activation or the pretreatment composition of the immune tumor microenvironment.

## Teaser

There remains a key knowledge gap in the application of novel immunotherapies in clinical practice, and more data are required to nominate clinicogenomic factors to prioritize future biomarker development. This retrospective analysis was conducted to provide a comprehensive analysis of the clinical and genomic predictors of response to immune checkpoint inhibitors in hepatocellular carcinoma.

**Implications for practice**

Although immune checkpoint inhibitor-based therapies have changed the treatment paradigm for advanced hepatocellular carcinoma, only a subset of patients respond with no clinical biomarkers to predict benefit. This study demonstrates that patient clinical factors such as performance status and liver function determine outcomes and that routine clinical grade genomics were not associated with outcomes.

## Introduction

Immune checkpoint inhibitors (ICIs), either in combination or as monotherapy, are now a mainstay of the treatment landscape for patients with hepatocellular carcinoma (HCC).^1-6^ Anti-PD-1/PD-L1 monotherapy leads to durable responses in a minor subset of patients with HCC, and in comparative studies, offers non-inferior overall survival (OS) when compared to sorafenib. Several anti-PD-1/L1 combinations therapies—atezolizumab plus bevacizumab, durvalumab with a priming dose of tremelimumab, and camrelizumab plus rivoceranib—result in superior OS when compared to sorafenib in treatment-naïve, patients with advanced HCC.^4-6^

Anti-PD1/PD-L1-based combination therapy in the 1st-line setting results in objective responses rates ranging for 20.1%-27.3%, median progression-free survival (PFS) ranging from 3.8 to 6.8 months, and median OS ranging from 16.4 to 22.1 months.^4-6^ The clinical trials that defined the operating characteristics of these effective combination regimens were largely restricted to physiologically fit patients with well-compensated liver function who do not necessarily represent patients in real-world clinical practice. Despite their established clinical utility, it is also important to acknowledge that a significant proportion of patients with HCC fail to respond to these therapies, necessitating the need for both predictive and prognostic markers to guide treatment selection and identify patients with a high risk of a poor outcome.

Response to ICI is influenced by a complex interplay between host factors, tumor characteristics, and the resultant immune response within the tumor microenvironment.^7^ Clinical parameters, including performance status, liver function, and etiologic factors, have also been evaluated as potential predictors of treatment response.^8-10^ Tumor genomic alterations are also posited to modulate the response to ICI including microsatellite instability high (MSI-H) status, tumor mutational burden (TMB), and WNT/β-catenin pathway activation.^11,12^ Finally, immune activation in the tumor microenvironment as assayed by PD-L1 expression, immune inflamed transcriptomic signatures, and immune cell infiltration, may also be predictive of ICI response.^9,11,13,14^

There remains a key knowledge gap in the application of novel immunotherapies in clinical practice, and more data are required to nominate clinicogenomic factors to prioritize future biomarker development. Improved patient selection has the potential to maximize therapeutic efficacy, minimize unnecessary toxicities, and ultimately enhance patient outcomes. We therefore conducted a retrospective analysis of the Memorial Sloan Kettering Cancer Center (MSK) clinical database with the primary objective of providing a comprehensive analysis of the clinical and genomic predictors of response to ICI in HCC.

## Materials and Methods

### Study design

This was a single-center, retrospective study of patients with advanced HCC treated with ICI with the primary objective to explore the impact of clinicopathologic and tumor genomic characteristics on outcome.

Patients were eligible if they had a histologically confirmed diagnosis of advanced HCC (unresectable Barcelona Center for Liver Cancer (BCLC) stage B or stage C) who received ≥ 1 dose of ICI with either anti-PD-1/L-1 therapy (nivolumab, pembrolizumab, atezolizumab, or durvalumab) or anti-CTLA-4 therapy (ipilimumab or tremelimumab) alone or in combination between May 2012 and April 2022. Fibrolamellar and mixed cholangiocarcinoma histologies, those patients who received concurrent regional therapy (ie, ablation, transarterial embolization, transarterial chemoembolization, transarterial radioembolization, or radiotherapy) for liver limited disease, and those patients with HCC with synchronous/metachronous malignancy were excluded.

Eligible patients were identified via query of an electronically maintained institutional database. Clinicopathologic features at the time of diagnosis and at the time that ICI therapy initiation were extracted including: data of birth; a history of hepatitis B virus (HBV)/hepatitis C virus (HCV) status; body mass index (BMI); Eastern Cooperation Oncology Group (ECOG) performance status (PS); BCLC stage; sites of disease (liver limited, extrahepatic disease, and large vascular involvement); tumor molecular profile; liver function panel; Child Pugh score; Albumin-Bilirubin (ALBI) score; alpha-fetoprotein (AFP); ICI type, line of therapy, and date of ICI start and end; date of investigator-assessed progression of disease; and date or death or last follow-up.

This study was approved by the MSK Institutional Review Board (IRB# 21-481) and was conducted in accordance with the U.S. Common Rule.

### Genomic analysis

Tumors and matched normal blood samples were analyzed using MSK-IMPACT, a clinically validated hybridization capture-based targeted next-generation sequencing array.^15^ MSK-IMPACT detects mutations, small insertions and deletions, copy number alterations, and select structural rearrangements. MSIsensor was used to evaluate MSI status, with tumors that had an MSIsensor score ≥ 10 being designated as MSI-H.^16^ Tumor mutational burden was calculated as the total number of nonsynonymous mutations divided by the number of bases analyzed. Genes were grouped into pathways using curated templates from the Cancer Genome Atlas (TCGA).^17^ OncoKB was used to filter variants of unknown significance.^18^ Different versions of the assay were used over the 10-year study period (341 genes, *n* = 3; 410, *n* = 16; 468, *n* = 49; 505, *n* = 23).

All samples were collected following written informed consent on an IRB-approved research protocol (MSK IRB 12-245; NCT01775072).

### Statistical analysis

Patient characteristics were summarized using descriptive statistics. Categorical data were summarized as frequencies with percentages and continuous data as medians and ranges. PFS was determined from the date of ICI initiation to the date of investigator-assessed progression or death, whichever occurred first. Patients alive without evidence of progression and who did not receive additional treatment by last follow-up were censored at last available date of radiological disease assessment. OS was estimated from the start of ICI until death. Patients alive at last follow-up were censored. Kaplan-Meier methods were used to estimate PFS and OS and visualize survival outcomes. Cox proportional hazards regression was used to assess univariate association with clinicopathologic and genomic factors at the time of ICI initiation with PFS and OS separately for patients treated in the 1st, 2nd, or≥3rd-line setting. Factors at *P* < .1 on univariable analyses were included in the multivariable Cox regression model. To avoid multicollinearity observed between BCLC stage, vascular involvement, and liver limited disease, only BCLC stage was included in the final PFS model for patients treated with ICI at the 1st -line. The multivariable model was not performed for patients treated with ICI in the ≥3rd line due to heterogeneity of patients and small number of patients in this group.

In order to explore additional genomic factors of response and resistance, additional oncogenic alteration, oncogenic signaling pathways, and fraction genome altered (FGA) were evaluated. For this analysis, patients were dichotomized into PFS < 12 months and PFS ≥ 12 months. Patients who were progression free but did not have 12-month follow-up were included in the PFS<12 months group. Fisher exact’ test was used to examine the association between genomic features and dichotomized PFS status on all patients, as well as separately within line of treatment.

All statistical analyses were performed using R Version 4.2.2 (R Foundation for Statistical Computing, Vienna, Austria). All *P-*values were two sided. *P-*values of <0.05 were considered to indicate statistical significance.

## Results

### Patient characteristics

From 2012 to 2022, 314 patients were identified from the database query; 72 patients were excluded based on mixed histology, fibrolamellar variant, concurrent local regional therapy, or synchronous/metachronous secondary malignancy (Supplementary Figure S1).

Two hundred forty-two patients with unresectable, transplant ineligible, or metastatic HCC were included in the analysis and their clinicopathologic characteristics at baseline are reported in Table 1 and at the time of initiation of ICI treatment in Table 2. Patients were predominantly male (80.7%) with a median age at diagnosis of 67 (range 23-94) years. A non-viral risk factor or no obvious risk factor was seen in 113 (47%) patients. At the time of ICI treatment, 213 (88%) had BCLC stage C disease, 120 (50%) patients had macrovascular involvement, and α-fetoprotein was ≥ 400 in 100 (41%) patients. Child-Pugh liver function was A in 170 (70%), and B in 72 (30%) patients. ALBI grade was 1 in 66 (27%), 2 in 151 (62%), and 3 in 26 (11%) patients. In the subset of patients (91/242; 38%) who underwent tissue-based molecular profiling the median TMB was 4.4 muts/Mb (range 0.8-98.4), and alterations in the WNT/β catenin pathway were noted in 40 (44%).

**Table 1.** Baseline patient characteristics at diagnosis.

Characteristic	Overall, *N* = 242^a^
*Sex*	
Female	46 (19%)
Male	196 (81%)
Age at diagnosis	67 (23, 94)
*Etiology*	
Hepatitis B	43 (18%)
Hepatitis C	86 (36%)
Non-viral	113 (47%)
*BCLC stage at diagnosis*	
A	48 (20%)
B	98 (40%)
C	96 (40%)
Systemic therapy prior to immunotherapy	152 (63%)
*Line of immunotherapy treatment*	
1	91 (38%)
2	123 (51%)
≥ 3	28 (11%)

^a^ *N* (%), median (range).

Abbreviation: BCLC, Barcelona clinic liver cancer.

**Table 2.** Patient characteristics at the time of immune checkpoint inhibitor-based therapy initiation.

Characteristic	Overall, *N* = 242^a^	ICI first line, *N* = 91^a^	ICI second line, *N* = 123^a^	ICI 3 + lines, *N* = 28^a^
*BCLC stage*				
B	29 (12%)	14 (15%)	14 (11%)	1 (3.6%)
C	213 (88%)	77 (85%)	109 (89%)	27 (96%)
Macrovascular involvement	120 (50%)	42 (46%)	67 (54%)	11 (39%)
Liver limited disease	78 (32%)	32 (35%)	41 (33%)	5 (18%)
Median AFP	210 (1, 1,179,130)	119 (1, 1,179,130)	236 (2, 623,309)	902 (4, 112,904)
*AFP*				
≥ 400	100 (41%)	31 (34%)	54 (44%)	15 (54%)
< 400	142 (59%)	60 (66%)	69 (56%)	13 (46%)
*Immunotherapy treatment*				
Combination	70 (29%)	49 (54%)	19 (15%)	2 (7.1%)
Single agent	172 (71%)	42 (46%)	104 (85%)	26 (93%)
Interval from diagnosis to immunotherapy treatment (months)	12 (0, 154)	7 (0, 154)	12 (1, 119)	27 (9, 135)
Median body mass index (BMI)	26.9 (16.9, 54.8)	27.9 (17.0, 40.9)	26.5 (16.9, 54.8)	24.5 (19.2, 40.0)
*BMI categories*				
Normal (18.5-24.9)	81 (33%)	19 (21%)	47 (38%)	15 (54%)
Overweight (> 25)	153 (63%)	69 (76%)	71 (58%)	13 (46%)
Underweight (< 18.5)	8 (3.3%)	3 (3.3%)	5 (4.1%)	0 (0%)
*ECOG performance status*				
0	31 (13%)	20 (22%)	10 (8.1%)	1 (3.6%)
1	179 (74%)	62 (68%)	95 (77%)	22 (79%)
2	32 (13%)	9 (9.9%)	18 (15%)	5 (18%)
Median serum albumin	3.50 (1.90, 4.50)	3.70 (2.20, 4.50)	3.40 (1.90, 4.50)	3.30 (2.60, 4.40)
*Serum albumin (g/dL)* ^b^				
< 3	44 (18%)	11 (12%)	26 (21%)	7 (25%)
≥ 3	198 (82%)	80 (88%)	97 (79%)	21 (75%)
*ALBI grade* ^c^				
G1	65 (27%)	36 (40%)	22 (18%)	7 (25%)
G2	151 (62%)	48 (53%)	83 (67%)	20 (71%)
G3	26 (11%)	7 (7.7%)	18 (15%)	1 (3.6%)
*Child-Pugh A vs B*				
A	170 (70%)	71 (78%)	78 (63%)	21 (75%)
B	72 (30%)	20 (22%)	45 (37%)	7 (25%)
Somatic tissue NGS	91 (38%)	32	42	17
Median TMB	4 (0.8-98.4)	3.5 (0.8-52.7)	4.4 (0.8-98.4)	3.3 (0.9-7.9)
WNT——pathway altered	40 (44%)	16 (50%)	17 (41%)	7 (41%)

^a^ *n* (%); median (range).

^b^Normal albumin range 3.8-5.0 g/dL.

^c^ALBI: albumin-bilirubin grade 1 = ≤ −2.60, 2 = > −2.60 to ≤ −1.39, 3 = > −1.39.

Abbreviations: ICI, immune checkpoint inhibitor; BCLC, Barcelona liver cancer centre; AFP, alpha fetoprotein; NGS, next generation sequencing; TMB, tumor mutational burden.

Ninety-one (38%) patients received an ICI in the 1st -line, 123 (51%) in 2nd line, and 28 (11%) in ≥ 3rd line. One hundred and seventy-two (71%) patients received single agent anti-PD-1/L1 or anti-CTLA-4 therapy, and 70 (29%) patients anti-PD-1/L1-based combination therapy (anti-VEGF therapy 44/70, 62%; anti-CTLA-4 therapy 10/70, 14%; and other 16/70, 24%). Median time on treatment for all patients was 2.3 months (0.0—-47.2).

### Cohort disposition and clinical outcomes

**Figure 1.**
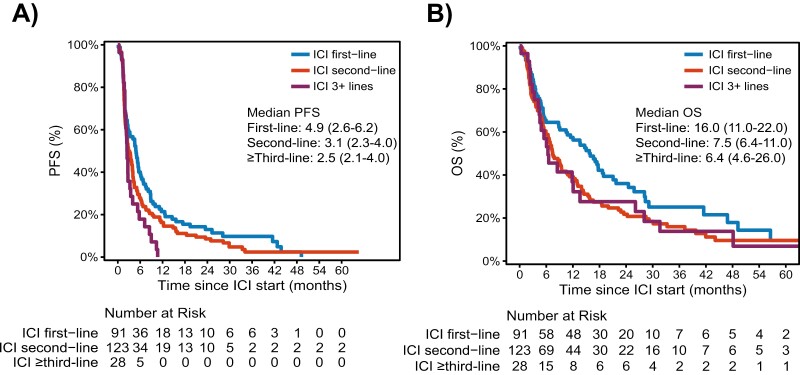
A) Progression free and B) overall survival from the time of anti-PD-1/L-1-based therapy initiation stratified by line of therapy given.

With a median follow-up time of 28 months (0.5-78.4) for the entire cohort, 188 (77%) deaths and 226 (93%) progression events were known to have occurred. Median PFS for the entire cohort was 3.4 (95% CI; 2.6-4.1) months and for those treated in 1st, 2nd, and ≥ 3rd-line was 4.9 (95% CI; 2.9-6.2), 3.1 (95% CI; 2.3-4.0), and 2.5 (95% CI; 2.1-4.0) months, respectively (Figure 1A). The 1- and 2-year estimated PFS in the 1st-line setting was 21% [95% CI: 14%-32%] and 13% [95%CI: 7.5%-23%] and for 2^nd^-line therapy was 16% [95%CI: 11%-24%] and 8.6% [95% CI: 4.8%-15%], respectively.

Median OS for those treated with ICI-based therapy in 1st, 2nd, and ≥ 3rd line was 16 (95% CI; 11-22), 7.5 (95% CI; 6.4-11), and 6.4 months (95% CI; 4.6-26), respectively (Figure 1B). The 1- and 2-year estimated OS in the 1st line was 57% [95%CI: 48%-69%], 38% [95%CI: 29%-50%], and for patients treated as 2nd line was 39% [95%CI: 31%-49%] and 22% [95%CI: 15%-31%], respectively.

Nine patients that had radiological progression of disease noted on single agent ICI were rechallenged with further ICI. Two patients received anti-PD-L1 plus anti-VEGF therapy with stable disease in 1 for 7.4 months. Anti-CTLA-4 was administered in combination with anti-PD-1 therapy in 5 patients with no responses with treatment duration ranging from 1.2 to 2.6 months. One patient received anti-PD-1 therapy in combination with a TKI with stable disease for 5.4 months. Two patients were rechallenged with single agent anti-PD-1 therapy following other therapy with no response noted in either patient.

#### Clinicogenomic factors associated with PFS

In univariate analysis, advanced BCLC stage, macrovascular involvement, extrahepatic disease, poorer ECOG-PS, and evidence of reduced liver function (Child-Pugh score B and ALBI grade 3) were all significantly associated with poorer PFS with ICI given in the 1^st^ line (Supplementary Figure S2 and Table S1). Extrahepatic disease, poorer ECOG PS, reduced liver function measured by Child-Pugh and ALBI score were significantly associated with shorter PFS with treatment given in 2nd line. The only clinical factor that was associated with a longer PFS in both 1st- and 2nd-line, was serum albumin ≥ 3. When compared to those with a non-viral etiology, a known history of hepatitis C virus was associated with improved PFS in 1st-line. In 3rd or greater lines, higher BMI appeared to be protective (Supplementary Table S1). There was no effect of the type of immunotherapy given, either single agent anti-PD-1/L1 or in combination with other agents, the serum AFP level, or WNT/βcatenin activated or TMB, on PFS, irrespective of what line of treatment immunotherapy was given (Supplementary Table S1).

**Figure 2.**
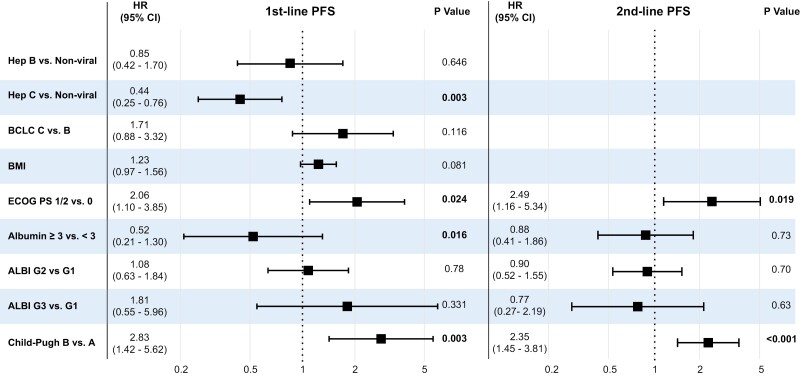
Forest plot of multivariate analysis of clinical factors associated with progression-free survival for patients treated with immunotherapy in the first and second line.

Multivariable analysis confirmed the significant association of worse ECOG PS and Child-Pugh score with reduced 1st line and 2nd line PFS, while BCLC stage C disease compared to stage B was also associated with increased risk of progression in 2nd line but did not reach statistical significance (Figure 2, Supplementary Table S2). A history of hepatitis C was associated with improved PFS compared to those with non-viral HCC in those treated in the 1st line.

#### Clinicogenomic factors associated with OS

In univariate analysis, advanced BCLC stage, macrovascular involvement, AFP ≥ 400, poorer ECOG-PS, increased BMI, and evidence of reduced liver function (Child-Pugh score B and ALBI grade 3) were all significantly associated with shorter OS in patients who received ICI as 1st-line systemic therapy (Supplementary Table S3). Vascular involvement, poorer ECOG PS, reduced liver function measured by Child-Pugh score and higher ALBI were significantly associated with shorter OS in patients treated with ICI as 2nd-line therapy. Serum albumin ≥ 3 was protective and associated with a longer OS across all lines of therapy, whereas a history of hepatitis C infection was associated with improved OS in patients treated with ICI in the 1st-line setting. Improved survival was observed with ICI combination therapy given in 1st- and 2nd -line, with a trend toward reduced survival when given in 3rd line. Genomic factors, such as WNT/β catenin activated or TMB, were not associated with OS, irrespective of the timing of immunotherapy treatment (Supplementary Table S3).

**Figure 3.**
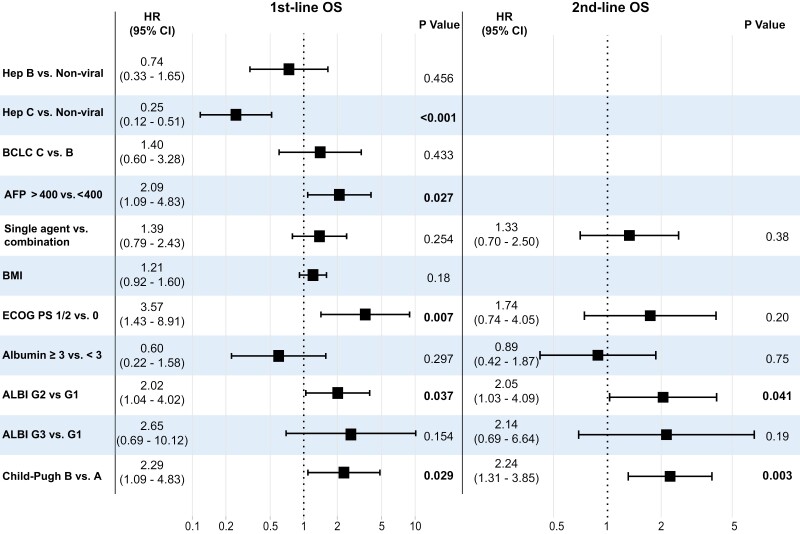
Forest plot of multivariate analysis of clinical factors associated with overall survival for patients treated with immunotherapy in the first and second line.

Multivariable analysis confirmed a significant association between increased AFP (≥ 400 ng/mL), poorer ECOG PS, ALBI grade 2 vs grade 1, and Child Pugh B liver function with reduced survival for those patients treated with ICI in 1st-line setting. Child-Pugh B compared to A and ALBI grade 2 vs grade 1, also significantly negatively impacted survival in those patients treated with ICI in the 1st- or 2nd-line setting (Figure 3, Supplementary Table S4). A known history of hepatitis C virus infection was also confirmed to be significantly associated with improved OS in patients treated with ICI in the 1st-line setting.

#### Exploratory genomic predictors of long-term disease control

**Figure 4.**
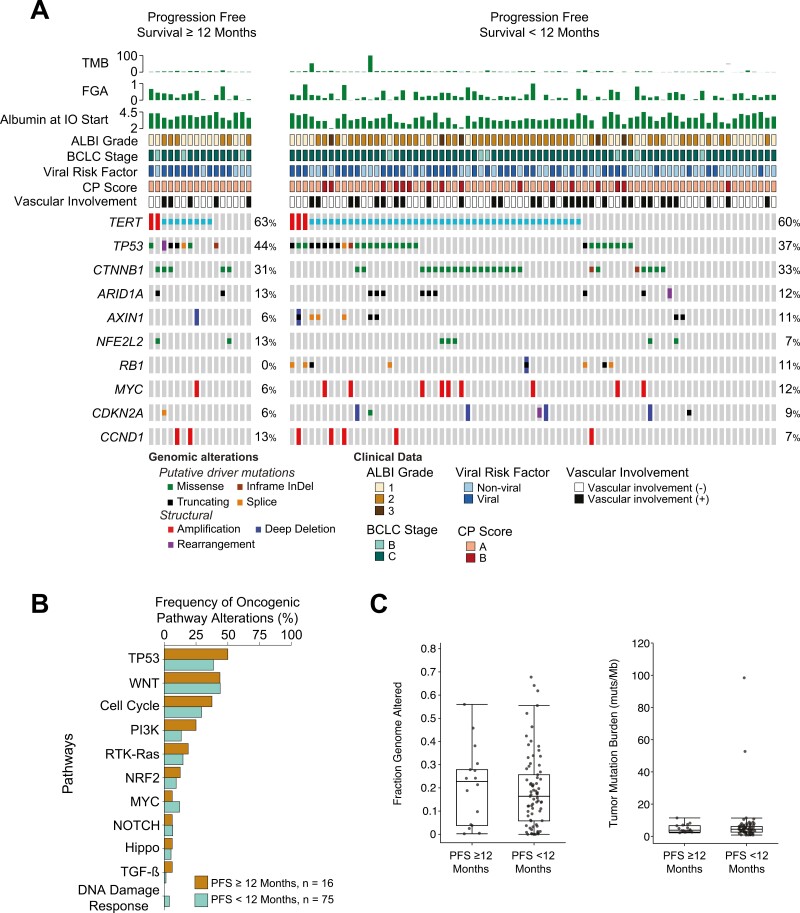
(A) Oncoprint of most frequently altered genes identified grouped depending on PFS < 12 months or ≥ 12 months. (B) Frequency of pathway-level alterations in PFS ≥ 12 months vs  < 12 months. (C) Fraction genome altered (FGA) and tumor mutational burden (TMB) in PFS ≥ 12 months vs < 12 months.

Ninety-one (38%) patients underwent tissue-based NGS sequencing with testing performed a median of 8.3 months prior to start of treatment. The average sequence coverage was 617X. Median TMB was 4.4 mut/Mb (range 0.8-98.4), FGA was 0.17, and no patients in the cohort had MSI-H disease. The genes with the highest frequency of oncogenic/likely oncogenic somatic mutations were *TERT* (55, 60%), *TP53* (35, 38%), and *CTNNB1* (30, 33%). Oncogenic *TERT*, *TP53*, *CTNNB1*, *AXIN1*, *RB1*, *MYC*, *CDKN2A* mutations were not predictive of PFS ≥ 12 months to ICI (Figure 4A). Furthermore, when mutations were grouped by pathway (TP53, WNT/β catenin, cell cycle, PI3K/AKT/mTOR, RTK-RAS, NRF2, MYC, NOTCH, Hippo, TGF-β, and DNA damage response; Figure 4B), TMB, or FGA (Figure 4C) no significant differences were observed between the patients that had a PFS ≥ 12 months compared to those with a PFS on ICI < 12 months.^17^ Analysis performed separately within line of ICI treatment also did not demonstrate any difference between these genomic features and the 2 PFS groups (data not shown).

## Discussion

In this large real-world cohort of patients with advanced HCC treated with ICI at a tertiary referral center, we observed that both PFS and OS in patients treated with ICI-based therapy were predominantly impacted by patient and disease specific factors. Impaired liver function, as assessed by Child Pugh or ALBI score, worse ECOG performance status, and clinical markers of greater disease burden were associated with worse progression-free and OS in HCC patients treated with ICI. Virally mediated HCC—specifically a history of HCV infection—was also associated with a favorable outcome, consistent with prior studies in other cancer types suggesting a higher likelihood of response in tumors with a viral etiology.^10,19,20^ In contrast to prior preclinical and clinical reports, somatic mutational profiles, specifically high TMB and mutations associated with WNT-β catenin activation, were not predictive of a favorable outcome in patients treated with ICI.^11,21-23^

Demographic characteristics of the MSK cohort were representative of the patients with HCC treated at a Center in the Western Hemisphere, although the study population was enriched with patients who had high-risk clinical features (ie, 50% macrovascular, ECOG PS ≥ 1 87%, and Child Pugh B 30%). Despite the inclusion of patients who would typically be excluded from clinical trials of novel ICI-based therapies, the median PFS and OS noted in our cohort was similar to those of recently reported prospective clinical trials in both the 1st and 2^nd^ line.^1,2,5,6^ Consistent with the natural history of HCC, we also observed a higher burden of disease, more extrahepatic disease, higher AFP, and worse liver function in patients who received ICI in later treatment lines. As such, it is not unexpected that PFS and OS were worse when administered in the 2nd- and 3rd-line setting.

Impaired liver function consistently emerged as a predictor of a poor outcome to ICI in the 1st and 2nd line in both univariate and multivariate analysis in our cohort. Given the critical role the liver plays in immune regulation, hepatic functional impairment and cirrhosis may compromise host immune response thereby affecting ICI efficacy and outcome.^24-26^ In addition, it is well established that disease burden, as well as liver function impact prognosis in patients with advanced HCC. Although subgroup analysis of recent prospective studies restricted to Child Pugh A population, indicate a benefit for immunotherapy irrespective of ALBI score (1 and 2), it remains unclear how ICIs operates in the setting of more advanced cirrhosis and decompensated liver function characterized by Child-Pugh B and C scores. Our data align with recent retrospective studies and metanalyses that liver function is a determinant of outcome to ICI in HCC, and further prospective study is required to confirm the benefit of ICI in this population.^27-32^

Recent preclinical data have suggested that non-alcoholic steatohepatitis-affected livers are more immunosuppressive, and HCCs that arise in this context are less sensitive to immunotherapy.^19^ Despite these observations, subset analyses in all prospective studies of immunotherapy as well as a recent larger metanalysis indicate that ICI is active in both virally and non-virally mediated HCC.^10,33^ Other clinical studies suggest a better outcome with ICI in patients with virally mediated disease.^34^ Following adjustment for confounders in multivariate analysis, patients with a known history of hepatitis C as a risk factor for the development of HCC appeared to have a more favorable response to ICI as well as prolonged survival compared to those with no viral hepatitis risk factor in our cohort. We also observed a trend toward improvement in patients with HBV-associated HCC. Further analyses of these observation are required, but given conflicting data in this regard, clinical practice should not rely on etiology as selection factor for ICI treatment.^35^

Prior reports suggested that patients with HCC harboring oncogenic alterations in WNT-β catenin had worse outcomes when treated with ICI monotherapy.^11,13,22,36,37^ Initial preclinical models and translational studies provided a mechanistic basis for this clinical association with immune exclusion implicated as a basis for insensitivity to anti-PD-1 therapy in WNT-driven cancers.^22,37^ Thus, a critical question is whether routine clinical tumor genomic profiling of patients with HCC might nominate a patient subset that is less responsive to immunotherapy. In the current series of nearly 100 molecularly profiled patients with HCC, we did not observe an association between WNT-β catenin pathway alterations and time-to-event survival analyses or in dichotomized analyses of those with a PFS ≥ 12 months compared to < 12 months on ICI. It is possible that biologic factors, not assessed in our study, such genomic heterogeneity, or study specific factors such as missing genomic data and the retrospective design of the current study may have impacted our ability detect an association between WNT pathway alterations and clinical outcome. Recent reports indicate that an inflamed immune microenvironment, which has been associated with respond to ICI, is not dependent on the underlying genomic drivers in HCC. For example, C*TNNB1* mutations characterized by weak WNT-β catenin signaling are associated with immune activation, whereas those that led to high level WNT-β catenin signaling are associated with an immune desertic TME phenotype.^38^ We also explored TMB, FGA as well other common gene and oncogenic pathway alterations in HCC, although none had impact on outcomes to ICI. Given that only 7% of the 91 patients with genomic data available had a high TMB (≥ 10 mut/Mb) it was not possible to formally assess PFS in this group compared to those with TMB < 10 mut/Mb. Our observations indicate that future studies should seek to explore differences in the immune microenvironment between responders and non-responders, a global effort that is being led by MSK.^39^ It is likely that a combination of clinical and immunologic features would likely provide the most accurate predictive and prognostic information for those treated with ICI in advanced HCC.^9,21,40^

A small subset of patients in our study had ICI rechallenging after prior progression on immunotherapy. Retrospective analyses demonstrated that post single-agent ICI failure, the ORR for addition of anti-CTLA-4 was 16% with 3 complete responses and a median time to progression of 2.96 months.^41^ These data are promising and suggest potential therapeutic utility of this approach, which should be studied further in prospective clinical trials.

Our study has several limitations: the single-center, retrospective, design; lack of a validation cohort; a high proportion of patients treated with anti-PD-1/L-1 monotherapy (which was largely due to the data collection period during ICI clinical development in HCC); missing data for our targeted next generation analyses; lack of tissue-based correlates to nominate other biomarkers.

## Conclusion

This cohort of histologically confirmed advanced HCC treated with ICI-based provides useful information in a more representative patient with HCC population and helps clarify the role of NGS. Consistently, irrespective of line or type of therapy received, the most critical factors impacting outcomes appear to relate to the patients clinical and functional status with no clear predictive genomic biomarkers at the gene or pathway level readily available from routine NGS.

## Supplementary material

Supplementary material is available at *The Oncologist* online.

oyae110_suppl_Supplementary_Figure_S1

oyae110_suppl_Supplementary_Figure_S2

oyae110_suppl_Supplementary_Table_S1

oyae110_suppl_Supplementary_Table_S2

oyae110_suppl_Supplementary_Table_S3

oyae110_suppl_Supplementary_Table_S4

## Data Availability

All data are available following request to corresponding author. Genomic data will be available for visualization on cBioPortal.org.

## References

[1] El-Khoueiry AB , Sangro B, Yau T, et al. Nivolumab in patients with advanced hepatocellular carcinoma (CheckMate 040): an open-label, non-comparative, phase 1/2 dose escalation and expansion trial. Lancet (London, England). 2017;389(10088):2492-2502. 10.1016/S0140-6736(17)31046-2 28434648 PMC7539326

[2] Yau T , Park J-W, Finn RS, et al. Nivolumab versus sorafenib in advanced hepatocellular carcinoma (CheckMate 459): a randomised, multicentre, open-label, phase 3 trial. Lancet Oncol. 2022;23(1):77-90. 10.1016/S1470-2045(21)00604-5 34914889

[3] Qin S , Kudo M, Meyer T, et al. LBA36 Final analysis of RATIONALE-301: Randomized, phase III study of tislelizumab versus sorafenib as first-line treatment for unresectable hepatocellular carcinoma. Ann Oncol. 2022;33(7):S1402-S1403. 10.1016/j.annonc.2022.08.033 30969136

[4] Qin S , Chan SL, Gu S, et al. Camrelizumab plus rivoceranib versus sorafenib as first-line therapy for unresectable hepatocellular carcinoma (CARES-310): a randomised, open-label, international phase 3 study. The Lancet. 2023;402(10408):1133-1146. 10.1016/S0140-6736(23)00961-3 37499670

[5] Abou-Alfa GK , Lau G, Kudo M, et al. Tremelimumab plus durvalumab in unresectable hepatocellular carcinoma. NEJM Evidence. 2022;1(8):EVIDoa2100070. 10.1056/EVIDoa2100070 38319892

[6] Finn RS , Qin S, Ikeda M, et al; IMbrave150 Investigators. Atezolizumab plus Bevacizumab in Unresectable Hepatocellular Carcinoma. N Engl J Med. 2020;382(20):1894-1905. 10.1056/NEJMoa1915745 32402160

[7] Sangro B , Sarobe P, Hervás-Stubbs S, Melero I. Advances in immunotherapy for hepatocellular carcinoma. Nat Rev Gastroenterol Hepatol. 2021;18(8):525-543. 10.1038/s41575-021-00438-0 33850328 PMC8042636

[8] Scheiner B , Pomej K, Kirstein MM, et al. Prognosis of patients with hepatocellular carcinoma treated with immunotherapy—development and validation of the CRAFITY score. J Hepatol. 2022;76(2):353-363. 10.1016/j.jhep.2021.09.035 34648895

[9] Sangro B , Melero I, Wadhawan S, et al. Association of inflammatory biomarkers with clinical outcomes in nivolumab-treated patients with advanced hepatocellular carcinoma. J Hepatol. 2020;73(6):1460-1469. 10.1016/j.jhep.2020.07.026 32710922 PMC7751218

[10] Meyer T , Galani S, Lopes A, Vogel A. Aetiology of liver disease and response to immune checkpoint inhibitors: An updated meta-analysis confirms benefit in those with non-viral liver disease. J Hepatol. 2023;79(2):e73-e76. 10.1016/j.jhep.2023.04.012 37086920

[11] Harding JJ , Nandakumar S, Armenia J, et al. Prospective genotyping of hepatocellular carcinoma: clinical implications of next-generation sequencing for matching patients to targeted and immune therapies. Clin Cancer Res. 2019;25(7):2116-2126. 10.1158/1078-0432.CCR-18-2293 30373752 PMC6689131

[12] Ang C , Klempner SJ, Ali SM, et al. Prevalence of established and emerging biomarkers of immune checkpoint inhibitor response in advanced hepatocellular carcinoma. Oncotarget. 2019;10(40):4018-4025. 10.18632/oncotarget.26998 31258846 PMC6592287

[13] Sia D , Jiao Y, Martinez-Quetglas I, et al. Identification of an Immune-specific Class of Hepatocellular Carcinoma, Based on Molecular Features. Gastroenterology. 2017;153(3):812-826. 10.1053/j.gastro.2017.06.007 28624577 PMC12166766

[14] Dai Y , Qiang W, Lin K, et al. An immune-related gene signature for predicting survival and immunotherapy efficacy in hepatocellular carcinoma. Cancer Immunol, Immunother: CII. 2021;70(4):967-979. 10.1007/s00262-020-02743-0 33089373 PMC10992402

[15] Cheng DT , Mitchell TN, Zehir A, et al. Memorial Sloan Kettering-Integrated Mutation Profiling of Actionable Cancer Targets (MSK-IMPACT): A Hybridization Capture-Based Next-Generation Sequencing Clinical Assay for Solid Tumor Molecular Oncology. J Mol Diagn. 2015;17(3):251-264. 10.1016/j.jmoldx.2014.12.006 25801821 PMC5808190

[16] Niu B , Ye K, Zhang Q, et al. MSIsensor: microsatellite instability detection using paired tumor-normal sequence data. Bioinformatics. 2014;30(7):1015-1016. 10.1093/bioinformatics/btt755 24371154 PMC3967115

[17] Sanchez-Vega F , Mina M, Armenia J, et al; Cancer Genome Atlas Research Network. Oncogenic signaling pathways in the cancer genome Atlas. Cell. 2018;173(2):321-337.e10. 10.1016/j.cell.2018.03.035 29625050 PMC6070353

[18] Chakravarty D , Gao J, Phillips SM, et al. OncoKB: a precision oncology knowledge base. JCO Precis Oncol. 2017;2017(1):PO.17.00011. 10.1200/PO.17.00011 28890946 PMC5586540

[19] Pfister D , Núñez NG, Pinyol R, et al. NASH limits anti-tumour surveillance in immunotherapy-treated HCC. Nature. 2021;592(7854):450-456. 10.1038/s41586-021-03362-0 33762733 PMC8046670

[20] Ding Z , Dong Z, Chen Z, et al. Viral status and efficacy of immunotherapy in hepatocellular carcinoma: a systematic review with meta-analysis. Front Immunol. 2021;12(1):733530. 10.3389/fimmu.2021.733530 34659220 PMC8511422

[21] Yoo S-K , Chowell D, Valero C, Morris LGT, Chan TA. Pre-treatment serum albumin and mutational burden as biomarkers of response to immune checkpoint blockade. NPJ Precis Oncol. 2022;6(1):23. 10.1038/s41698-022-00267-7 35393553 PMC8990074

[22] Ruiz de Galarreta M , Bresnahan E, Molina-Sánchez P, et al. β-catenin activation promotes immune escape and resistance to anti–PD-1 therapy in hepatocellular carcinoma. Cancer Discovery. 2019;9(8):1124-1141. 10.1158/2159-8290.CD-19-0074 31186238 PMC6677618

[23] Luke JJ , Bao R, Sweis RF, Spranger S, Gajewski TF. WNT/β-catenin pathway activation correlates with immune exclusion across human cancers. Clin Cancer Res. 2019;25(10):3074-3083. 10.1158/1078-0432.CCR-18-1942 30635339 PMC6522301

[24] Albillos A , Lario M, Álvarez-Mon M. Cirrhosis-associated immune dysfunction: Distinctive features and clinical relevance. J Hepatol. 2014;61(6):1385-1396. 10.1016/j.jhep.2014.08.010 25135860

[25] Robinson MW , Harmon C, O’Farrelly C. Liver immunology and its role in inflammation and homeostasis. Cell Mol Immunol. 2016;13(3):267-276. 10.1038/cmi.2016.3 27063467 PMC4856809

[26] Llovet JM , Castet F, Heikenwalder M, et al. Immunotherapies for hepatocellular carcinoma. Nat Rev Clin Oncol. 2022;19(3):151-172. 10.1038/s41571-021-00573-2 34764464

[27] Vogel A , Frenette C, Sung M, et al. Baseline liver function and subsequent outcomes in the phase 3 REFLECT study of patients with unresectable hepatocellular carcinoma. Liver Cancer. 2021;10(5):510-521. 10.1159/000516490 34721512 PMC8527908

[28] Lee P-C , Chao Y, Chen M-H, et al. Predictors of response and survival in immune checkpoint inhibitor-treated unresectable hepatocellular carcinoma. Cancers. 2020;12(1):182. 10.3390/cancers12010182 31940757 PMC7017111

[29] Kuo Y-H , Wang J-H, Hung C-H, et al. Albumin-Bilirubin grade predicts prognosis of HCC patients with sorafenib use. J Gastroenterol Hepatol. 2017;32(12):1975-1981. 10.1111/jgh.13783 28295594

[30] Abdel-Rahman O. Impact of baseline characteristics on outcomes of advanced HCC patients treated with sorafenib: a secondary analysis of a phase III study. J Cancer Res Clin Oncol. 2018;144(5):901-908. 10.1007/s00432-018-2610-z 29455421 PMC11813483

[31] Xie E , Yeo YH, Scheiner B, et al. Immune checkpoint inhibitors for Child-Pugh Class B advanced hepatocellular carcinoma: a systematic review and meta-analysis. JAMA Oncol. 2023;9(10):1423-1431. 10.1001/jamaoncol.2023.3284 37615958 PMC10450588

[32] Kudo M , Matilla A, Santoro A, et al. CheckMate 040 cohort 5: a phase I/II study of nivolumab in patients with advanced hepatocellular carcinoma and Child-Pugh B cirrhosis. J Hepatol. 2021;75(3):600-609. 10.1016/j.jhep.2021.04.047 34051329

[33] Espinoza M , Muquith M, Lim M, et al. Disease etiology and outcomes after atezolizumab plus bevacizumab in hepatocellular carcinoma: post-hoc analysis of IMbrave150. Gastroenterology. 2023;165(1):286-288.e4. 10.1053/j.gastro.2023.02.042 36894034

[34] Haber PK , Puigvehí M, Castet F, et al. Evidence-based management of hepatocellular carcinoma: systematic review and meta-analysis of randomized controlled trials (2002–2020). Gastroenterology. 2021;161(3):879-898. 10.1053/j.gastro.2021.06.008 34126063 PMC12276942

[35] Pinto E , Meneghel P, Farinati F, et al. Efficacy of immunotherapy in hepatocellular carcinoma: Does liver disease etiology have a role? Dig Liver Dis. 2023;56(4):579-588. 10.1016/j.dld.2023.08.062 37758610

[36] Haber PK , Castet F, Torres-Martin M, et al. Molecular markers of response to anti-PD1 therapy in advanced hepatocellular carcinoma. Gastroenterology. 2023;164(1):72-88.e18. 10.1053/j.gastro.2022.09.005 36108710 PMC12182972

[37] Spranger S , Bao R, Gajewski TF. Melanoma-intrinsic β-catenin signalling prevents anti-tumour immunity. Nature. 2015;523(7559):231-235. 10.1038/nature14404 25970248

[38] Montironi C , Castet F, Haber PK, et al. Inflamed and non-inflamed classes of HCC: a revised immunogenomic classification. Gut. 2023;72(1):129-140. 10.1136/gutjnl-2021-325918 35197323 PMC9395551

[39] Faraj W , Robson M, Tawil A, et al. Biospecimen repositories in low- and middle-income countries: insights from an American University of Beirut and Memorial Sloan Kettering Collaboration. JCO Global Oncol. 2023;9(9):e2300140. 10.1200/GO.23.00140 PMC10846789 37883726

[40] Zhu AX , Abbas AR, de Galarreta MR, et al. Molecular correlates of clinical response and resistance to atezolizumab in combination with bevacizumab in advanced hepatocellular carcinoma. Nat Med. 2022;28(8):1599-1611. 10.1038/s41591-022-01868-2 35739268

[41] Wong JSL , Kwok GGW, Tang V, et al. Ipilimumab and nivolumab/pembrolizumab in advanced hepatocellular carcinoma refractory to prior immune checkpoint inhibitors. J ImmunoTher Cancer. 2021;9(2):e001945. 10.1136/jitc-2020-001945 33563773 PMC7875295

