# Supplementary material for: Clinicogenomic predictors of outcomes in patients with hepatocellular carcinoma treated with immunotherapy
Source: Oncologist. 2024 Jun 27;29(10):894–903. doi: 10.1093/oncolo/oyae110 (PMC11448888; doi:10.1093/oncolo/oyae110)
Supplement: oyae110_suppl_Supplementary_Figure_S1 [file oyae110_suppl_supplementary_figure_s1.docx]

**Figure S1:** Consort schema of patients included for evaluation

**
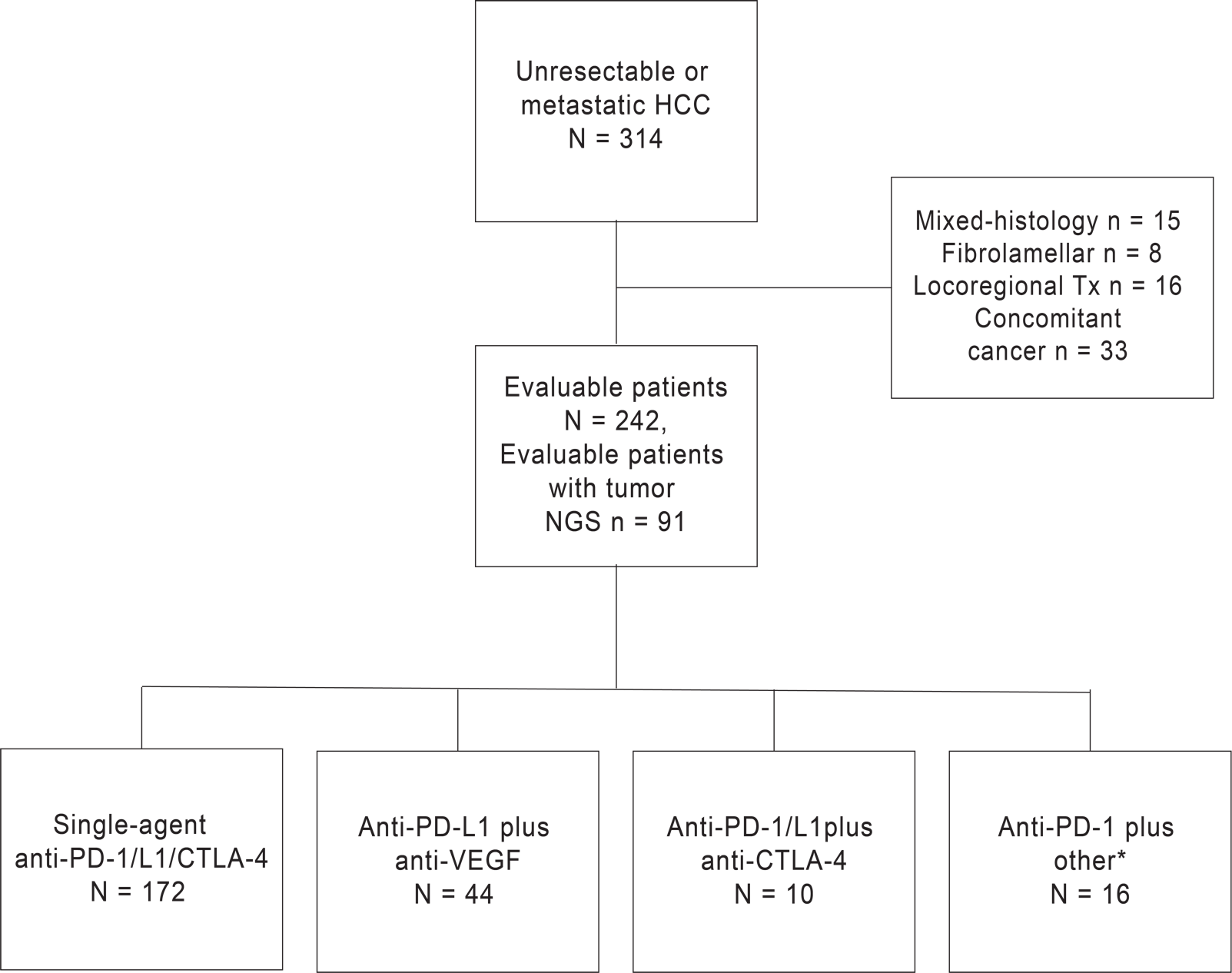
**

*other therapies given in combination with anti-PD-1/L1 therapy included tyrosine kinase inhibitors (n=13) and investigational agent (n=2) and chemotherapy (n=1)
