# Supplementary material for: Clinicogenomic predictors of outcomes in patients with hepatocellular carcinoma treated with immunotherapy
Source: Oncologist. 2024 Jun 27;29(10):894–903. doi: 10.1093/oncolo/oyae110 (PMC11448888; doi:10.1093/oncolo/oyae110)
Supplement: oyae110_suppl_Supplementary_Figure_S2 [file oyae110_suppl_supplementary_figure_s2.docx]

**Figure S2:** Progression-free survival in first and second line stratified by select clinical factors

**
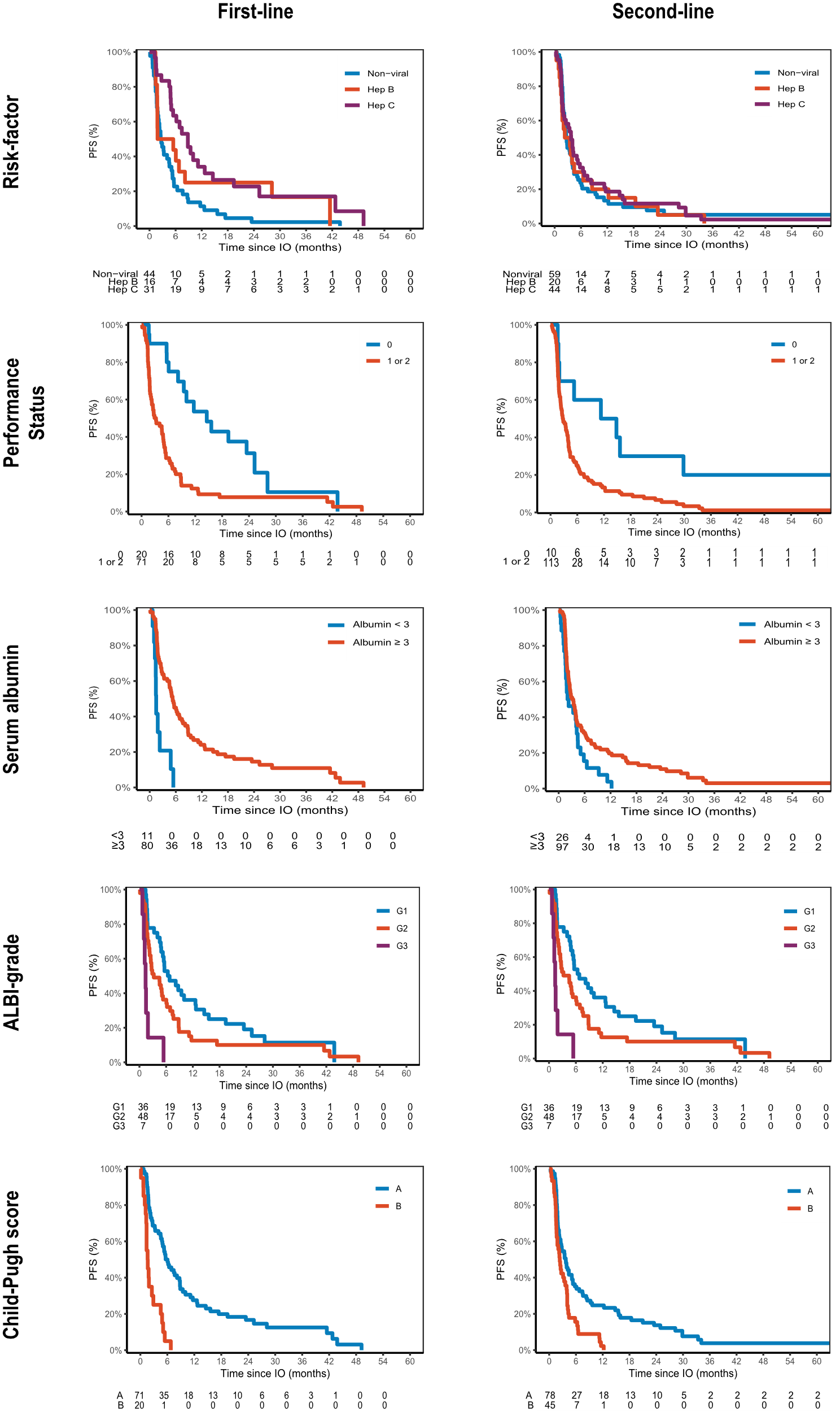
**
