# Supplementary material for: Clinicogenomic predictors of outcomes in patients with hepatocellular carcinoma treated with immunotherapy
Source: Oncologist. 2024 Jun 27;29(10):894–903. doi: 10.1093/oncolo/oyae110 (PMC11448888; doi:10.1093/oncolo/oyae110)
Supplement: oyae110_suppl_Supplementary_Table_S1 [file oyae110_suppl_supplementary_table_s1.docx]

**Table S1**: Univariate analysis of patient and disease characteristics association with immunotherapy progression-free survival. Bold figures indicate statistical significance.

|  |  | **1^st^ line** |  |  | **2^nd^ line** |  |  | **≥3^rd^ line** |  |
| --- | --- | --- | --- | --- | --- | --- | --- | --- | --- |
| **Characteristic** | **HR**^a^ | **95% CI**^a^ | **p-value** | **HR**^a^ | **95% CI**^a^ | **p-value** | **HR**^1^ | **95% CI**^a^ | **p-value** |
| **Disease factors** |  |  |  |  |  |  |  |  |  |
| Etiology |  |  |  |  |  |  |  |  |  |
| Non-viral | — | — |  | — | — |  | — | — |  |
| Hep B | 0.62 | 0.34, 1.14 | 0.12 | 1.02 | 0.61, 1.70 | 0.95 | 1.16 | 0.44, 3.09 | 0.76 |
| Hep C | 0.42 | 0.26, 0.70 | **<0.001** | 0.88 | 0.59, 1.32 | 0.55 | 0.76 | 0.30, 1.88 | 0.55 |
|  |  |  |  |  |  |  |  |  |  |
| BCLC^b^ stage |  |  |  |  |  |  |  |  |  |
| B | — | — |  | — | — |  | — | — |  |
| C | 2.10 | 1.11, 3.99 | **0.023** | 1.47 | 0.83, 2.63 | 0.19 | 1.94 | 0.26, 14.6 | 0.52 |
|  |  |  |  |  |  |  |  |  |  |
| Vascular involvement* | 1.81 | 1.16, 2.81 | **0.009** | 1.22 | 0.84, 1.75 | 0.29 | 0.96 | 0.44, 2.09 | 0.92 |
|  |  |  |  |  |  |  |  |  |  |
| Liver limited disease | 0.58 | 0.36, 0.92 | **0.022** | 0.58 | 0.39, 0.87 | **0.008** | 1.24 | 0.46, 3.33 | 0.67 |
|  |  |  |  |  |  |  |  |  |  |
| AFP^c^** | 1.00 | 1.00, 1.00 | 0.17 | 1.00 | 1.00, 1.00 | 0.43 | 1.00 | 1.00, 1.00 | 0.091 |
| AFP ≥ 400 | - | - |  | - | - |  | - | - |  |
| AFP < 400 | 0.98 | 0.61, 1.57 | 0.94 | 0.87 | 0.60, 1.25 | 0.45 | 1.4 | 0.64, 3.05 | 0.40 |
|  |  |  |  |  |  |  |  |  |  |
| Immunotherapy treatment |  |  |  |  |  |  |  |  |  |
| Combination | — | — |  | — | — |  | — | — |  |
| Single agent | 1.20 | 0.77, 1.87 | 0.42 | 1.48 | 0.88, 2.47 | 0.14 | 0.66 | 0.15, 2.84 | 0.57 |
|  |  |  |  |  |  |  |  |  |  |
| Patient clinical factors |  |  |  |  |  |  |  |  |  |
| Body mass index** | 1.04 | 1.00, 1.09 | 0.065 | 1.00 | 0.98, 1.03 | 0.78 | 0.92 | 0.84, 1.00 | **0.043** |
|  |  |  |  |  |  |  |  |  |  |
| ECOG Performance Score |  |  |  |  |  |  |  |  |  |
| 0 | — | — |  | — | — |  | — | — |  |
| 1 / 2 | 2.60 | 1.49, 4.54 | **<0.001** | 2.45 | 1.18, 5.07 | **0.016** | 1.71 | 0.23, 12.8 | 0.60 |
|  |  |  |  |  |  |  |  |  |  |
| Serum albumin** | 0.47 | 0.29, 0.75 | **0.002** | 0.74 | 0.54, 1.01 | 0.055 | 0.38 | 0.16, 0.91 | **0.030** |
| Albumin < 3 | - | - |  | - | - |  | - | - |  |
| Albumin ≥ 3 | 0.25 | 0.12, 0.51 | **<0.001** | 0.61 | 0.39, 0.95 | **0.030** | 0.41 | 0.16, 1.04 | 0.059 |
|  |  |  |  |  |  |  |  |  |  |
| ALBI grade |  |  |  |  |  |  |  |  |  |
| G1 | — | — |  | — | — |  | — | — |  |
| G2 | 1.56 | 0.98, 2.48 | 0.062 | 1.40 | 0.84, 2.34 | 0.19 | 1.60 | 0.66, 3.90 | 0.30 |
| G3 | 6.22 | 2.63, 14.7 | **<0.001** | 2.11 | 1.08, 4.10 | **0.028** | 1.42 | 0.17, 12.0 | 0.75 |
|  |  |  |  |  |  |  |  |  |  |
| Child-Pugh score |  |  |  |  |  | **<0.001** |  |  |  |
| A | — | — |  | — | — |  | — | — |  |
| B | 4.24 | 2.42, 7.43 | **<0.001** | 1.99 | 1.34, 2.95 |  | 3.02 | 1.15, 7.92 | **0**.**025** |
|  |  |  |  |  |  |  |  |  |  |
| Genomic factors |  |  |  |  |  |  |  |  |  |
| TMB^d^ (N=33,42, 13) | 1.01 | 0.97, 1.05 | 0.65 | 1.00 | 0.98, 1.03 | 0.77 | 1.32 | 0.97, 1.80 | 0.080 |
| WNT pathway altered (N=31, 42, 14) | 1.85 | 0.84, 4.05 | 0.12 | 0.86 | 0.46, 1.60 | 0.63 | 1.23 | 0.40, 3.77 | 0.71 |
| ^a^HR: Hazard Ratio, CI: 95% Confidence Interval, ^b^BCLC: Barcelona liver cancer center, ^c^AFP: alpha fetoprotein, ^d^TMB: tumour mutational burden. | | | | | | | | | |
