# Supplementary material for: Clinicogenomic predictors of outcomes in patients with hepatocellular carcinoma treated with immunotherapy
Source: Oncologist. 2024 Jun 27;29(10):894–903. doi: 10.1093/oncolo/oyae110 (PMC11448888; doi:10.1093/oncolo/oyae110)
Supplement: oyae110_suppl_Supplementary_Table_S2 [file oyae110_suppl_supplementary_table_s2.docx]

**Table S2**: Multivariate analysis of factors found to be statistically significant in univariate analysis and their impact on progression-free survival on immunotherapy-based treatment in 1^st^ and 2^nd^ line. Bold figures indicate statistical significance.

| **MV PFS 1^st^ line** | | | | **MV PFS 2^nd^ line** | | |
| --- | --- | --- | --- | --- | --- | --- |
| **Characteristic** | **HR**^a^ | **95% CI**^a^ | **p-value** | **HR**^a^ | **95% CI**^a^ | **p-value** |
| Etiology |  |  |  | — |  |  |
| Non-viral | — | — |  |  |  |  |
| Hep B | 0.85 | 0.42, 1.70 | 0.65 |  |  |  |
| Hep C | 0.44 | 0.25, 0.76 | **0.003** |  |  |  |
| BCLC stage |  |  |  | — |  |  |
| B | — | — |  |  |  |  |
| C | 1.71 | 0.88, 3.32 | 0.12 |  |  |  |
| Body mass index* | 1.23 | 0.97, 1.56 | 0.081 | — |  |  |
| Performance status |  |  |  |  |  |  |
| 0 | — | — |  | — |  |  |
| 1 / 2 | 2.06 | 1.10, 3.85 | **0.024** | 2.49 | 1.16,5.34 | **0.019** |
|  |  |  |  |  |  |  |
| Albumin < 3 | - | - |  |  |  |  |
| Albumin ≥ 3 | 0.52 | 0.21, 1.30 | 0.16 | 0.88 | 0.41, 1.86 | 0.73 |
|  |  |  |  |  |  |  |
| ALBI grade |  |  |  |  |  |  |
| G1 | — | — |  | — | — |  |
| G2 | 1.08 | 0.63, 1.84 | 0.78 | 0.90 | 0.52, 1.55 | 0.70 |
| G3 | 1.81 | 0.55, 5.96 | 0.33 | 0.77 | 0.27, 2.19 | 0.63 |
|  |  |  |  |  |  |  |
| Child-Pugh score |  |  |  |  |  |  |
| A | — | — |  | — | — |  |
| B | 2.83 | 1.42, 5.62 | **0.003** | 2.35 | 1.45,3.81 | **<0.001** |
| ^a^HR = Hazard Ratio, CI = Confidence Interval; * HR compared per 1-unit increase in the continuous variable. | | | | | | |
