# Supplementary material for: Clinicogenomic predictors of outcomes in patients with hepatocellular carcinoma treated with immunotherapy
Source: Oncologist. 2024 Jun 27;29(10):894–903. doi: 10.1093/oncolo/oyae110 (PMC11448888; doi:10.1093/oncolo/oyae110)
Supplement: oyae110_suppl_Supplementary_Table_S3 [file oyae110_suppl_supplementary_table_s3.docx]

**Table S3**: Univariate analysis of clinical and disease characteristics associated with overall survival. Bold figures indicate statistical significance.

|  |  | **1^st^ line** |  |  | **2^nd^ line** |  |  | **≥3^rd^ line** |  |
| --- | --- | --- | --- | --- | --- | --- | --- | --- | --- |
| **Characteristic** | **HR**^a^ | **95% CI**^a^ | **p-value** | **HR**^a^ | **95% CI**^a^ | **p-value** | **HR**^a^ | **95% CI**^a^ | **p-value** |
| **Disease factors** |  |  |  |  |  |  |  |  |  |
| Etiology |  |  |  |  |  |  |  |  |  |
| Non-viral | — | — |  | — | — |  | — | — |  |
| Hep B | 0.53 | 0.27, 1.05 | 0.068 | 0.83 | 0.47, 1.47 | 0.53 | 1.48 | 0.52, 4.26 | 0.47 |
| Hep C | 0.48 | 0.27, 0.86 | **0.013** | 0.82 | 0.53, 1.27 | 0.38 | 0.67 | 0.26, 1.77 | 0.42 |
|  |  |  |  |  |  |  |  |  |  |
| BCLC stage |  |  |  |  |  |  |  |  |  |
| B | — | — |  | — | — |  | — | — |  |
| C | 2.62 | 1.17, 5.84 | **0.019** | 1.14 | 0.63, 2.04 | 0.67 | 2.75 | 0.34, 21.9 | 0.34 |
|  |  |  |  |  |  |  |  |  |  |
| Vascular involvement * | 2.21 | 1.33, 3.68 | **0.002** | 1.68 | 1.13, 2.51 | **0.011** | 1.41 | 0.61, 3.29 | 0.42 |
|  |  |  |  |  |  |  |  |  |  |
| Liver limited disease | 0.62 | 0.36, 1.07 | 0.085 | 0.74 | 0.49, 1.13 | 0.16 | 1.02 | 0.34, 3.07 | 0.98 |
|  |  |  |  |  |  |  |  |  |  |
| AFP** | 1.00 | 1.00, 1.00 | **0.011** | 1.00 | 1.00, 1.00 | 0.79 | 1.00 | 1.00, 1.00 | 0.12 |
| AFP < 400 | - | - |  | - | - |  | - | - |  |
| AFP ≥ 400 | 1.65 | 0.99, 2.76 | 0.056 | 1.11 | 0.74, 1.64 | 0.62 | 0.81 | 0.35, 1.90 | 0.63 |
|  |  |  |  |  |  |  |  |  |  |
| Immunotherapy treatment |  |  |  |  |  |  |  |  |  |
| Combination | — | — |  | — | — |  | — | — |  |
| Single agent | 1.79 | 1.08, 2.97 | **0.024** | 1.85 | 1.03, 3.32 | **0.039** | 0.49 | 0.11, 2.14 | 0.34 |
|  |  |  |  |  |  |  |  |  |  |
| Patient clinical factors |  |  |  |  |  |  |  |  |  |
| BMI* | 1.05 | 1.00, 1.11 | **0.04** | 1.00 | 0.97, 1.03 | 0.85 | 0.92 | 0.83, 1.02 | 0.01 |
|  |  |  |  |  |  |  |  |  |  |
| Performance score |  |  |  |  |  |  |  |  |  |
| 0 | — | — |  | — | — |  | — | — |  |
| 1 / 2 | 5.44 | 2.33, 12.7 | **<0.001** | 2.43 | 1.06, 5.55 | **0.036** | 1.97 | 0.26, 15.1 | 0.51 |
|  |  |  |  |  |  |  |  |  |  |
| Serum albumin ** | 0.32 | 0.19, 0.52 | **<0.001** | 0.49 | 0.35, 0.68 | **<0.001** | 0.21 | 0.07, 0.59 | **0.003** |
| Albumin < 3 | - | - |  | - | - |  | - | - |  |
| Albumin ≥ 3 | 0.24 | 0.11, 0.50 | **<0.001** | 0.47 | 0.29, 0.76 | **0.002** | 0.30 | 0.11, 0.84 | **0.022** |
|  |  |  |  |  |  |  |  |  |  |
| ALBI Grade |  |  |  |  |  |  |  |  |  |
| G1 | — | — |  | — | — |  | — | — |  |
| G2 | 2.60 | 1.48, 4.57 | **<0.001** | 2.76 | 1.45, 5.23 | **0.002** | 2.43 | 0.82, 7.22 | 0.11 |
| G3 | 11.8 | 4.55, 30.6 | **<0.001** | 5.48 | 2.47, 12.2 | **<0.001** | 0.00 | 0.00, Inf | >0.99 |
|  |  |  |  |  |  |  |  |  |  |
| Child-Pugh Score |  |  |  |  |  |  |  |  |  |
| A | — | — |  | — | — |  | — | — |  |
| B | 3.89 | 2.2, 7.02 | **<0.001** | 3.36 | 2.16, 5.21 | **<0.001** | 4.10 | 1.45, 11.6 | **0.008** |
|  |  |  |  |  |  |  |  |  |  |
| Genomic factors |  |  |  |  |  |  |  |  |  |
| TMB (N = 33,42,13) | 0.97 | 0.87, 1.08 | 0.57 | 1.01 | 0.98, 1.03 | 0.48 | 0.97 | 0.74, 1.27 | 0.81 |
|  |  |  |  |  |  |  |  |  |  |
| WNT pathway altered (N = 31, 42, 14) | 1.00 | 0.38, 2.61 | 0.99 | 0.94 | 0.47, 1.87 | 0.85 | 0.81 | 0.24, 2.68 | 0.72 |
| ^a^HR = Hazard Ratio, CI = Confidence Interval; *HR compared per 1-unit increase in the continuous variable | | | | | | | | | |
