# Supplementary material for: Clinicogenomic predictors of outcomes in patients with hepatocellular carcinoma treated with immunotherapy
Source: Oncologist. 2024 Jun 27;29(10):894–903. doi: 10.1093/oncolo/oyae110 (PMC11448888; doi:10.1093/oncolo/oyae110)
Supplement: oyae110_suppl_Supplementary_Table_S4 [file oyae110_suppl_supplementary_table_s4.docx]

**Table S4**: Multivariate analysis of clinical and disease characteristics associated with overall survival from the time of anti-PD-1/L-1 based therapy initiation

|  |  | **MV OS 1^st^ line** |  | **MV OS 2^nd^ line** | | |
| --- | --- | --- | --- | --- | --- | --- |
| **Characteristic** | **HR**^a^ | **95% CI**^a^ | **p-value** | **HR**^a^ | **95% CI**^a^ | **p-value** |
|  |  |  |  |  |  |  |
| Etiology |  |  |  | — | — |  |
| Non-viral | — | — |  |  |  |  |
| Hep B | 0.74 | 0.33, 1.65 | 0.46 |  |  |  |
| Hep C | 0.25 | 0.12, 0.51 | **<0.001** |  |  |  |
|  |  |  |  |  |  |  |
| BCLC stage |  |  |  | — |  |  |
| B | — | — |  |  |  |  |
| C | 1.40 | 0.60, 3.28 | 0.43 |  |  |  |
|  |  |  |  |  |  |  |
|  |  |  |  |  |  |  |
| AFP < 400 | — | — |  | — |  |  |
| AFP ≥ 400 | 2.09 | 1.09, 4.02 | **0.027** |  |  |  |
|  |  |  |  |  |  |  |
| Immunotherapy treatment |  |  |  |  |  |  |
| Combination | — | — |  | — | — |  |
| Single agent | 1.39 | 0.79, 2.43 | 0.25 | 1.33 | 0.70,2.50 | 0.38 |
|  |  |  |  |  |  |  |
| BMI* | 1.21 | 0.92, 1.6 | 0.18 |  |  |  |
|  |  |  |  |  |  |  |
| Performance status |  |  |  |  |  |  |
| 0 | — | — |  | — | — |  |
| 1 or 2 | 3.57 | 1.43, 8.91 | **0.007** | 1.74 | 0.74, 4.05 | 0.20 |
|  |  |  |  |  |  |  |
| Albumin < 3 | — | — |  | — | — |  |
| Albumin ≥ 3 | 0.60 | 0.22, 1.58 | 0.30 | 0.89 | 0.42, 1.87 | 0.75 |
|  |  |  |  |  |  |  |
| ALBI grade |  |  |  |  |  |  |
| G1 | — | — |  | — | — |  |
| G2 | 2.02 | 1.04, 3.91 | **0.037** | 2.05 | 1.03, 4.09 | **0.041** |
| G3 | 2.65 | 0.69, 10.1 | 0.15 | 2.14 | 0.69, 6.64 | 0.19 |
|  |  |  |  |  |  |  |
| Child Pugh Score |  |  |  |  |  |  |
| A | — | — |  | — | — |  |
| B | 2.82 | 1.31, 6.05 | **0.008** | 2.24 | 1.31, 3.85 | **0.003** |
|  |  |  |  |  |  |  |
| ^a^HR = Hazard Ratio, CI = Confidence Interval; *HR compared per 1-unit increase in the continuous variable | | | | | | |
